# Hypertension artérielle maligne en milieu néphrologique à Abidjan: à propos de 168 cas colligés au Service de Néphrologie-Médecine Interne du Centre Hospitalier Universitaire de Treichville

**DOI:** 10.11604/pamj.2021.38.305.21303

**Published:** 2021-03-24

**Authors:** Jean Astrid Aka, Cyr Monlet Guei, Serge Didier Konan, Patrick Sery Diopoh, Syndou Sanogo, Hubert Kouamé Yao

**Affiliations:** 1 Service de Néphrologie - Médecine Interne D, Centre Hospitalier Universitaire de Treichville, Boulevard de Marseille, Abidjan, Côte d´Ivoire,; 2 Service de Néphrologie, Centre Hospitalier Universitaire de Yopougon, Abidjan, Côte d’Ivoire

**Keywords:** Hypertension artérielle maligne, insuffisance rénale chronique, rétinopathie, hypertrophie ventriculaire gauche, accident vasculaire cérébral, Malignant arterial hypertension, chronic renal failure, retinopathy, left ventricular hypertrophy, stroke

## Abstract

L´hypertension artérielle maligne (HTAM) est une entité nosologique peu décrite en milieu néphrologique. L´objectif était de décrire le profil des patients présentant une HTA maligne en milieu néphrologique et identifier les facteurs pronostiques. Étude rétrospective, descriptive et analytique réalisée de janvier 2013 à décembre 2018 dans le service de néphrologie du CHU de Treichville. Le diagnostic d´HTA maligne était retenu devant une pression artérielle diastolique (PAD) ≥ 130 mmHg, une rétinopathie hypertensive au stade III ou IV selon Keith Wegener, avec une ou plusieurs autres atteintes viscérales cardiaques et/ou cérébrales et/ou rénales. Nous avons colligé 168 patients. L´âge moyen était de 41,10 ± 14,86 ans avec une prédominance masculine (sex-ratio de 1,54). Les facteurs de risque cardiovasculaire étaient l´HTA (79,20%), l´alcool (32,10%), le tabac (19,60%), la maladie rénale chronique (15,30%) et le diabète (11,30%). Ils étaient admis pour dyspnée (39,29%), poussée hypertensive (26,16%), troubles de la conscience (10 ,12%). L´examen clinique notait une anémie (82,10%), des œdèmes des membres inférieurs (63,10%), un œdème aigu du poumon (37,50%). Pour le retentissement de l´HTA, nous avons observé une insuffisance rénale (95,9%), une hypertrophie ventriculaire gauche (92,81%), un accident vasculaire cérébral (16,67%), un double retentissement cardiaque et rénal (85%). L´insuffisance rénale était chronique dans 78%. Les étiologies de l´HTAM étaient l´HTA essentielle (56,8%), la glomérulonéphrite chronique (29,8%) et le diabète (6%). L´évolution a été favorable dans 66,7% des cas et le décès constaté dans 25,6% des cas. En analyse multivariée, le taux d´urémie ≥ 2g/l [OR=5,07; IC95% = (2,39-10,75); p = 0,0001], l´hyperkaliémie [OR = 3,50; IC95% = (1,70-7,19); p = 0,001], l´hyponatrémie [OR = 2,90;IC95% = (1,40-6,03); p = 0,004], le taux d´hémoglobine < 12g/dl [OR = 5,91; IC 95% = (1,34-26,00); p = 0,019] et l´IR terminale [OR =6,06; IC95% = (2,04-18,18); p = 0,001] étaient les facteurs associés à la survenue de décès. L´HTA maligne est consécutive à une HTA mal ou non traitée. Elle touche l´adulte jeune avec des complications multiviscérales dominées dans notre contexte par l´insuffisance rénale chronique terminale (IRCT). D´où l´intérêt d´un diagnostic précoce et d´une prise en charge adéquate de toute HTA.

## Abstract (en)

**Introduction:**

malignant arterial hypertension (MAH) is a nosologic disorder which has not been described in Nephrology. The purpose of this study was to describe the profile of patients with MAH in the Division of Nephrology and to identify prognostic factors.

**Methods:**

we conducted a retrospective, descriptive and analytical study from January 2013 to December 2018 in the Unit of Nephrology of the University Hospital Center in Treichville. The diagnosis of MAH was retained in patients with diastolic blood pressure (DBP) ≥ 130 mmHg, Keith Wegener grade III/IV hypertensive retinopathy, one or multiple visceral, cardiac and/or brain and/or renal diseases.

**Results:**

we collected data from 168 patients. The average age of patients was 41.10 ± 14.86 years, with male predominance (sex ratio 1.54). Cardiovascular risk factors were AH (79.20%), alcohol (32.10%), tobacco (19.60%), chronic kidney disease (15.30%) and diabetes (11.30%). They were admitted with dyspnea (39.29%), hypertensive crisis (26.16%), consciousness disorders (10.12%). Clinical examination showed anemia (82.10%), lower limb edema (63.10%), acute pulmonary edema (37.50%). Arterial hypertension resulted in renal failure (95,9%), left ventricular hypertrophy (92.81%), stroke (16,67%), and cardiac and renal involvement (85%). Renal failure was chronic in 78% of cases. The causes of MAH were essential AH (56,8%), chronic glomerulonephritis (29.8%), and diabetes (6%). Outcome was favorable in 66,7% of cases and overall mortality rate was 25.6%. In multivariate analysis uremia ≥ 2g/l [OR=5,07; 95%CI = 2,39-10.75; p = 0.0001], hperkalaemia [OR = 3.50; 95% CI = 1.70 - 7.19; p = 0.001], hyponatremia [OR = 2.90; 95% CI= 1.40 - 6.03; p = 0.004], haemoglobin level < 12g/dl [OR=5,91; 95% CI=1,34-26,00; p=0,019] and end-stage renal disease [OR = 6.06; 95% CI = 2.04 - 18.18; p = 0.001] were factors associated with the occurrence of death.

**Conclusion:**

MAH is a consequence of poorly treated or untreated AH. It mainly affects young adults with multivisceral complications. In our Hospital, these were dominated by end-stage chronic renal disease. Hence the importance of early diagnosis and adequate management in patients with AH.

## Introduction

L´hypertension artérielle (HTA) est définie comme une élévation des chiffres tensionnels supérieure ou égale à 140 mmHg pour la pression artérielle systolique (PAS) et ou supérieure ou égale à 90 mmHg pour la pression artérielle diastolique (PAD) [1]. Elle constitue un véritable problème de santé publique [2] de par sa prévalence élevée, sa mortalité et ses complications évolutives: cardiaques, oculaires, cérébrales et rénales source d´handicaps, et son coût pour l´individu et la société. Elle représente la première cause de mortalité cardiovasculaire [3]. L´HTA se présente sous diverses formes dont certaines sont de véritables urgences médicales. L´une d´elles est l´hypertension artérielle maligne (HTAM) qui peut se développer de novo ou compliquer une HTA essentielle ou secondaire. L´HTAM associe une pression artérielle diastolique (PAD) supérieure ou égale à 130 mmHg à une rétinopathie hypertensive stade III (hémorragies, exsudats) ou IV (œdème papillaire) de la classification de Keith et Wegener, une ou plusieurs autres atteintes viscérales: cardiaques, cérébrales, rénales [4]. L´incidence de l´HTAM est en nette régression dans les pays développés. En Grande Bretagne, Lip *et al*. trouvait une incidence de l´HTAM entre 5 à 6 cas pour 100.000 habitants [5]. Cette prévalence reste élevée dans les pays en voies de développement. Ainsi, en Côte d´Ivoire, Odi Assamoi en milieu hospitalier cardiologique retrouvait une prévalence de 17,4% en 1989 [6] et elle était de 10,35% au Togo en 1995 selon Baragou *et al*. [7]. En milieu néphrologique, peu d´études ont été menées sur l´HTA maligne. Notre étude avait donc pour objectif de décrire le profil des patients présentant une HTAM en milieu néphrologique et d´identifier les facteurs pronostiques.

## Méthodes

### Lieu de l´étude

L´étude a été menée dans le service de néphrologie du CHU de Treichville. Ce service est constitué d´une unité de consultation, d´un hôpital du jour, d´une unité de prise en charge des patients infectés par le VIH et d´une unité d´hospitalisation conventionnelle.

### Population d´étude

Était inclus, tout patient âgé d´au moins 15 ans et hospitalisé pour HTA maligne. Les patients ayant un dossier médical incomplet pour les paramètres étudiés n´ont pas été inclus.

### Type et période d´étude

Il s´agit d´une étude rétrospective à visée descriptive et analytique sur une durée de 5 ans allant de janvier 2013 à décembre 2018.

### Recueil des données

Pour chaque patient inclus, les données suivantes ont été collectées à l´aide d´une fiche d´enquête standardisée: données épidémiologiques (âge, sexe, profession, domicile), données anamnestiques (notion d´œdèmes, d´hypertension artérielle (HTA), maladie rénale chronique (MRC), tabagisme, alcoolisme, sédentarité), données cliniques (motif d´admission, pression artérielle à l´admission, état de conscience, œdème des membres inférieurs (OMI), œdème aigue pulmonaire (OAP), anémie clinique, syndrome pyramidal, fond d´œil); données paracliniques (urée plasmatique, taux de créatinine sérique avec débit de filtration glomérulaire (DFG) estimé, protéinurie des 24 heures, hémogramme, ionogramme sanguin, calcémie, glycémie, échographie cardiaque, échographie rénale, électrocardiogramme (ECG); données évolutives (évolution favorable, décès, sortie contre avis médical, évolution de la pression artérielle, et de la fonction rénale.

### Définition des termes opérationnels

L´HTA maligne a été évoquée devant l´association d´une pression artérielle diastolique ≥ 130mmHg, une rétinopathie hypertensive au stade III ou IV de la classification de Keith et Wegener avec une ou plusieurs autres atteintes viscérales: cardiaques et /ou rénales et / ou cérébrales [4]. La maladie rénale chronique est définit par la persistance pendant au moins trois mois de marqueurs d´atteinte rénale ou de baisse du DFG < 60ml/min. Elle a été classée en 5 stades de gravité croissante selon la classification K/DIGO [8]. L´équation « Modification of Diet in Renal Disease » (MDRD) a été utilisée pour l´estimation du DFG.

Le caractère chronique de l´insuffisance rénale a été évoqué en présence de critères anamnestiques: antécédent de maladie rénale, antériorité de créatininémie élevée, présence ancienne d´une protéinurie ou d´anomalies du sédiment urinaire (hématurie, leucocyturie) et/ou de critères morphologiques: diminution de la taille des reins à l´échographie rénale et/ou de critères biologiques: anémie normochrome normocytaire, hypocalcémie. L´anémie est définie par un taux d´hémoglobine < 12g/dl. Elle a été considérée comme sévère pour un taux d´hémoglobine < 8g/dl et modérée pour un taux d´hémoglobine compris entre 8 et 11g/dl. L´hypocalcémie est définie par une diminution du taux de calcium sanguin < 85mg/l. L´hyponatrémie a été retenue pour un taux de sodium sanguin < 135meq/l et l´hyperkaliémie pour un taux de potassium > 5 meq/l. Pour le diabète, ont été considérés comme diabétiques, les patients connus diabétiques, et/ou ceux dont le diagnostic de diabète a été retenu pendant l´hospitalisation avec une glycémie à jeun > 1,26g/l à au moins deux dosages successifs ou une glycémie casuelle ≥ 2 g/l [9].

Les patients qui ont présenté un indice de masse corporelle (IMC) ≥ 30 Kg/m^2^ ont été considérés comme obèses. L´hypertrophie ventriculaire gauche (HVG) électrique a été définie à partir de l´indice de Sokolow-Lyon > 35 mm. La cardiomégalie a été retenue pour un index cardio-thoracique > 0,50 à la radiographie pulmonaire. La rétinopathie a été classée en quatre stades selon Keith et Wegener: stade I (rétrécissement artériolaire focal ou diffus); stade II (stade I + signe du croisement); stade III (stade II + hémorragies ou exsudats rétiniens); stade IV (stade III + œdème papillaire). Les troubles neurologiques comportaient les troubles de la conscience, les crises convulsives, l´agitation et le déficit hémicorporel. L´HTA a été classée en grades selon la classification OMS [10]. L´HTA a été dite essentielle, lorsqu´aucune étiologie n´a été retrouvée après les explorations cliniques et paracliniques. La glomérulonéphrite chronique a été retenue en présence d´une protéinurie abondante (> 1,5g/24heures) associée ou non à une hématurie, avec ou sans insuffisance rénale chronique.

### Saisie et analyse des données

Les données ont été saisies dans une base Excel et analysées à l´aide du logiciel « SPSS V22 ». Nous avons effectué une analyse univariée et multivariée, les variables quantitatives ont été décrites avec les moyennes ± écart type. Le test statistique X^2^ (Chi2) a été utilisé pour comparer les variables qualitatives. L´association entre la variable et la mortalité a été appréciée par l´odds ratio (OR). Le seuil de p < 0,005 était considéré comme statistiquement significatif.

## Résultats

Durant la période d´étude, nous avons colligé 168 cas d´HTA maligne. L´âge moyen de nos patients était de 41,10 ± 14,86 ans avec des extrêmes de 15 et de 85 ans. Nous avons observé une prédominance masculine avec un sex-ratio de 1,54. Les principales comorbidités étaient l´HTA (79,2%), la maladie rénale chronique (15,5%) et le diabète (11,3%). Les principaux signes cliniques étaient, en dehors de l´HTA de grade 3 observée chez tous les patients, une pâleur (82,1%), des œdèmes des membres inférieurs (63,1%) et un œdème aigu du poumon (37,5%) (Tableau 1).

**Tableau 1:** caractéristiques générales des patients avec HTA maligne

Variables	Total (n=168)	Décédés (n=43)	Vivants (n=125)	Valeur de p	OR (IC=95%)
**Sexe**					
Masculin	60,7% (102/168)	60,4% (26/43)	60,8%(16/125)	0 ,55	1 ,01 (0,59-1,71)
Féminin	39,3% (66/168)	39,5%(17/43)	39,2%(49/125)		
**Age (ans)**					
<35	29,8% (50/168)	37,2% (16/43)	27,2% (34/125)	0,14	1,39 (0,82-2,35)
[35-65[	58,9% (99/168)	48,8%(21/43)	62,4% (78/125)	0,84	0,66 (0,39-1,11)
≥ 65	11,3% (19/168)	14%(6/43)	10,4% (13/125)	0,35	1,27 (0,62-2,60)
**Comorbidités**					
HTA	79,2% (133/168)	86,05% (37/43)	76,8% (96/125)	0,14	0,61 (0,28-1,34)
Diabète	11,3% (19/168)	9,03% (4/43)	12% (15/125)	0,43	1,24 (0,50-3,09)
MRC	14,8% (25/168)	20,9% (9/43)	12,8% (16/125)	0,37	
Obésité	1,8% (3/168)	4,7% (2/43)	0,8%(1/125)	0,16	2,68 (1,15-6,23)
**Signes cliniques**					
Œdèmes	63,1% (106/168)	62,8% (27/43)	63,2%(79/125)	0,55	0,01 (0,59-1,72)
OAP	37,5% (63/168)	34,9% (15/43)	38 ,4% (48/125)	0,41	1,12 (0,65-1,92)
Pâleur	82,1% (138/168)	95,3% (41/43)	77,6%(97/125)	0,005	0,22 (0,05-0,87)
**Hémoglobine (g /dl)**					
Moyenne	8,96±2,51	7,77± 2,13	9,40± 2,49	0,0001	
>12	16,07% (27/168)	4,7% (2/43)	20% (25/125)	0,01	0,25 (0,06-0,99)
[8-12[	45,2% (76/168)	39,5% (17/43)	47,2% (59/125)	0,24	0,79 (0,46-1,34)
< 8	36,9% (62/168)	55,8% (24/43)	30,4% (38/125)	0,03	2,16 (1,29-3,61)
**Autres Signes biologiques**					
Hyperkaliémie	41,8% (64/153)	60,5% (26/43)	30,4% (38/125)	0,01	2,48(1,46-4,20)
Hyponatrémie	52,9% (81/153)	67,4% (29/43)	41,6% (52/125)	0,03	2,22 (1,26-3,90)
Hypocalcémie	55,3% (72/130)	51,2% (22/43)	40%(50/125)	0,13	1,39 (0,83-2,33)
Thrombopénie	38,4% (63/164)	39,5% (17/43)	36,8% (46/125)	0,44	1,09 (0,64-1,84)
**Etiologies HTAM**					
HTA essentielle	56,5% (95/168)	46,5% (20/43)	60% (75/125)	0,87	0,66 (0,93-1,11)
GNC	29,8% (50/168)	39,5% (17/43)	26,4% (33/125)	0,78	1,54 (0,92-2,58)
Diabète	6% (10/168)	7% (3/43)	5,6% (7/125)	0,49	1,18 (0,44-3,17)
Indéterminée	5,4% (9/168)	4,7% (2/43)	5,6% (7/125)	0,58	0,86 (0,24-3)
Autres	2,4% (4/168)	2,3%(1/43)	2,4%(3/125)	0,75	0,97(0,17-5,43)

OAP=Œdème Aigu du poumon ; MRC= Maladie rénale chronique ; GNC= Glomérulonéphrite chronique

Du point de vue du retentissement de l´HTA maligne, l´insuffisance rénale (IR) a été observée dans 95,9% des cas dont 78% d´IR chronique (Tableau 2). Cette IR était au stade terminal dans 53,1%. Tous les patients ont présenté une rétinopathie hypertensive dont 51,2% au stade 3, 47% au stade 4 et 1,8% l´association rétinopathie hypertensive au stade 3 + rétinopathie diabétique. L´HVG a été observée dans 92,8% des cas et la cardiomégalie dans 85,6% des cas. Les principales anomalies à l´échocardiographie étaient la cardiomyopathie dilatée (22,6%). L´accident vasculaire cérébral (AVC) a été observé chez 16,6% des patients. Nous avons observé un double retentissement cardiaque et rénal dans 85,5% des cas. L´anémie a été observée chez 83,6% des patients et celle-ci était sévère dans 37,5%. Les autres anomalies biologiques étaient l´hyperkaliémie (41,8%), l´hyponatrémie (52,9%), l´hypocalcémie (55,3%) et la thrombopénie (38,4%). Les principales étiologies de l´HTAM étaient l´HTA essentielle (56,5%), la glomérulonéphrite chronique (GNC) (29,8%), le diabète (6%).

**Tableau 2:** répartition selon le retentissement de l´HTA maligne

Retentissement	Total (n=168)	Décédés (n=43)	Vivants (n=125)	Valeur de p	OR (IC=95%)
**Insuffisance rénale**					
Aigue	14,3% (24/168)	4,7% (2/43)	17,6% (22/125)	0,26	0,29 (0,07-1,13)
Chronique	78% (131/168)	90,7% (39/43)	73,6% (92/125)	0,13	2,75 (1,05-7,20)
Indéterminée	3,6% (5/168)				
**Rétinopathie HTA**					
Stade III	51,2% (86/168)	44,2% (19/43)	53,6% (67/125)	0,28	
Stade IV	47% (79/168)	55,8% (24/43)	44%(55/125)		
**Cardiopathie**					
HVG	76,8% (129/168)	83,7% (36/43)	74,4% (93/125)	0,14	1,55 (0,75-3,21)
CMD	22,6% (38/168)	20,9% (9/43)	23,2% (29/125)	0,47	0,90 (0,47-1,71)
**Cérébral**					
AVC	16,6% (13/78)	11,6% (4/43)	7,2% (9/125)	0,28	1,44 (0,65-3,16)

HVC=Hypertrophie ventriculaire gauche; CMD = Cardiomyopathie dilatée; AVC= Accident vasculaire cérébral.

**Figure 1:**
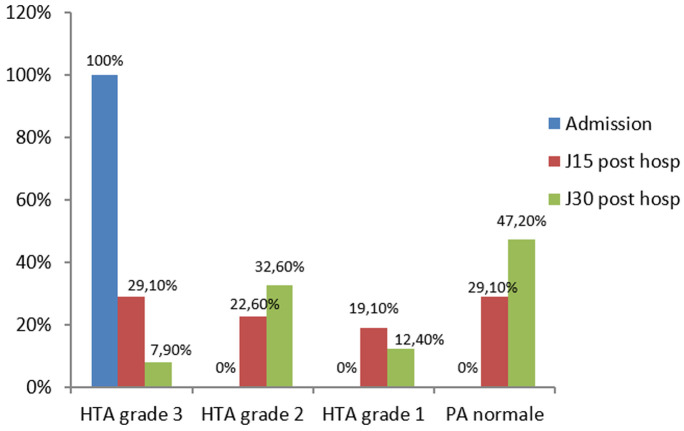
évolution de la pression artérielle 30 jours après l´hospitalisation

**Figure 2:**
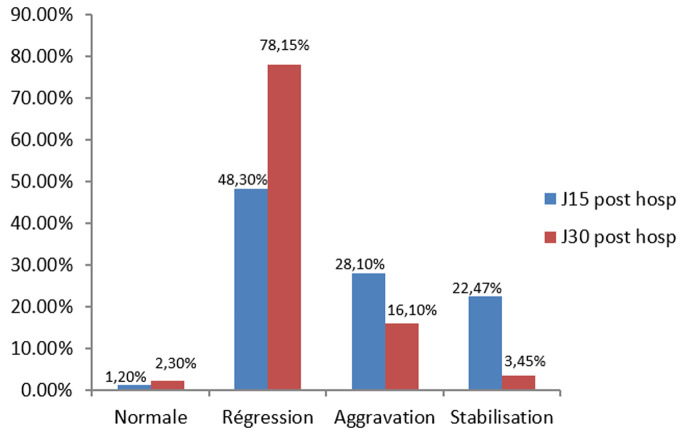
évolution de la fonction rénale 30 jours après l´hospitalisation

Les inhibiteurs calciques ont été les médicaments anti hypertenseurs les plus utilisés (65,5%). L´hémodialyse a été indiquée chez 54,9% des patients et a été effectivement réalisée dans 31,4% des cas. L´évolution a été favorable dans 66,7%. Nous avons observé une normalisation progressive de la pression artérielle chez 47% des patients à 30 jours post-hospitalisation (Figure 1). De même, une régression de l´insuffisance rénale a été observée dans 78,1% des cas à 30 jours post-hospitalisation (Figure 2).

La mortalité hospitalière était de 25,6%. En analyse univariée, la pâleur (p = 0,005), l´anémie (p = 0,01), le taux d´hémoglobine < 8g/dl (p = 0,03), l´hyperkaliémie (p = 0,01), l´hyponatrémie (p = 0,03) étaient associés au risque de décès (Tableau 3). De même, l´insuffisance rénale terminale était associée au risque de décès [OR= 4,3; IC 95% = 1,64-11,59; p = 0,0001]. En analyse multivariée, par régression logistique, les facteurs suscités étaient associés au risque de décès chez nos patients (Tableau 3).

**Tableau 3:** analyse multivariée par régression logistique

Variables	Valeur de p	OR (IC 95%)
Urée >2g/l	0,0001	5,07(2,39-10,78)
DFG<15ml/min	0,001	6,06(2,04-18,18)
Hyperkaliémie>5mEq/l	0,001	3,5(1,70-7,19)
Hyponatrémie<135mmol/l	0,004	2,90(1,40-6,03)
Hémoglobine<12g/dl	0,019	5,91(1,34-16)
Hémoglobine<8g/dl	0,003	2,89(1,41-5,89)
Aggravation de fonction rénale à M1	0,0001	9,46(2,70-13,10)

DFG= Débit de filtration glomérulaire; M1= à un mois.

## Discussion

Nos patients étaient relativement jeunes. En effet, 61,6% des patients avaient moins de 45 ans. Van den Born *et al*. au Pays-Bas [11], Herbland *et al*. en France [12] et Lengani *et al*. au Burkina Faso [13] trouvaient respectivement un âge moyen de 44 ± 12 ans, 43 ± 11 ans et 38 ± 4 ans. Houman *et al*. en Tunisie [14] notait un âge moyen de 55 ans. D´une façon générale, l´HTAM touche l´adulte jeune corroborant le constat de Baragou *et al*. [7]. Elle constitue le mode de découverte de l´HTA du sujet jeune [12]. Le sexe masculin était le plus prédominant dans notre série, tout comme dans celles de Lengani *et al*. [13] et Houman *et al*. [14] avec respectivement un sex-ratio de 2,7 et 3,2. Globalement, la prédominance masculine a été observée dans toutes les séries [15, 16]. Cette prédominance masculine pourrait être liée à la prévalence élevée de l´HTA chez l´homme.

Dans notre série, près de huit patients sur dix étaient hypertendus connus. Kadiri *et al*. au Nigéria [17] et Van Den Born *et al*. au Pays bas [11] notaient respectivement 33,8% et 58% de patients hypertendus connus. La dyspnée était le principal motif d´hospitalisation dans notre étude, suivi des troubles neurologiques. Lengani *et al*. [13] notait également une prédominance de la dyspnée avec une fréquence de 47,2%. Les céphalées étaient retrouvées chez 8,3% et le coma chez 5,6% des patients. Houman *et al*. [14] avait noté les céphalées chez 68,2% et les troubles de la conscience chez 42,1% des patients. Herbland *et al*. [12] retrouvait chez 40% des patients, la poussée hypertensive comme le motif de découverte de l´HTAM. Les céphalées sont donc fréquemment décrites dans la littérature comme motif de découverte de l´HTAM [16, 18]. Dans notre contexte, cette différence observée s´expliquerait par le fait que la majorité de nos patients consultaient aux stades des complications évolutives et les symptômes de gravité liée aux complications font que les céphalées sont relayées au second plan.

La présence de la rétinopathie hypertensive au stade avancé fait partie de la définition de l´HTA maligne. Ainsi, tous les patients présentaient une rétinopathie hypertensive au stade III ou IV. Herbland *et al*. [12] a observé une rétinopathie hypertensive au stade III chez 54,8% et au stade IV dans 45,3%. Dans l´étude de Houman *et al*. la rétinopathie était au stade III dans 57,9% et au stade IV dans 28,4%. Boni *et al*. [19] notait dans une population de patients avec une HTA sévère, une rétinopathie hypertensive au stade III dans 29% des cas et au stade IV dans 64% des cas. Nos résultats étaient en accord avec ceux de la littérature [4, 16, 18] qui trouvaient un fond d´œil au stade III et IV dans 100% des cas.

L´insuffisance rénale a été observée dans la quasi-totalité des cas. Nos résultats étaient proches de ceux de Lengani *et al*. [13] et Houman *et al*. [14] qui trouvaient une insuffisance rénale respectivement dans 94,4% et 100%. Herbland *et al*. [12] a retrouvé une insuffisance rénale dans 64,5%. L´insuffisance rénale était chronique chez 2/3 des patients. Toutes ces études ont été menées dans des services de néphrologie, ce qui expliquerait la proportion élevée de l´insuffisance rénale qui constitue donc le principal motif d´hospitalisation dans ces services. Globalement la proportion d´insuffisance rénale dans l´HTAM varie entre 70 et 100% des cas [18].

Dans notre étude, l´HVG était retrouvée chez plus de neuf patients sur dix. Nos résultats étaient proches de ceux de Lengani *et al*. [13] qui retrouvait une HVG chez 94,4% des patients. Herbland *et al*. [12] a observé une HVG dans 56% des cas. L´HVG est un marqueur péjoratif de l´HTA dans la survenue des complications. Plus l´HVG est présente, plus les risques d´évènements cardiovasculaires et de décès deviennent importants [20, 21]. La cardiomyopathie dilatée était la principale anomalie à l´écho Doppler cardiaque révélée sous la forme d´une insuffisance ventriculaire gauche type OAP observée chez un tiers des patients dans notre série. Nos résultats étaient en accord avec ceux de la littérature qui notaient une insuffisance cardiaque dans 20-40% des cas [15].

Dans notre étude, un AVC a été observé chez 13 patients soit 16,67%. Odi Assamoi [6] retrouvait un AVC dans 15% des cas en milieu cardiologique. Cette faible fréquence d´AVC pourrait s´expliquer par le fait que notre étude ayant été menée dans un service de néphrologie, la plupart des patients présentant un déficit neurologique sont évacués dans des services spécialisés notamment ceux de neurologie et de réanimation. Il est admis que l´HTAM augmenterait de façon considérable le risque d´AVC. Le facteur précipitant serait l´augmentation rapide de la pression artérielle entrainant une perte de l´autorégulation vasomotrice des vaisseaux cérébraux [16]. Dans notre série, plus de huit patients sur dix présentaient une anémie qui était sévère dans plus d´un tiers des cas. Elle est d´une part hémolytique mécanique liée aux lésions de microangiopathie thrombotique et d´autre part liée à la sévérité de l´insuffisance rénale responsable d´un défaut de synthèse d´érythropoïétine. Cette MAT s´accompagne habituellement de thrombopénie observée chez un tiers de nos patients. Des lésions endothéliales liées à l´élévation de la pression artérielle dans l´HTAM sont responsables de la survenue de la MAT dans ce contexte [16].

Les autres anomalies étaient représentées essentiellement par l´hypocalcémie, l´hyponatrémie et l´hyperkaliémie. Elles sont le reflet de l´IRC au stade avancé observé chez nos patients. L´hyponatrèmie résulte de la déplétion sodée due à la natriurèse de pression observée dans l´HTAM. Cependant dans notre contexte, elle pourrait s´expliquer par une hémodilution. En effet, 63,10% de nos patients présentaient un syndrome œdémateux. L´HTA essentielle représentait la cause principale de l´HTAM dans notre étude. Yao *et al*. dans une étude portant sur la multithérapie antihypertensive ont observé 60% d´HTA essentielle [22]. Dans l´étude de Lip *et al*. [5] à Birmingham (Angleterre), l´HTA essentielle représentait 56,4% des causes d´HTAM. Globalement, l´HTAM survient dans la majorité des cas, chez des patients ayant une HTA essentielle préexistante mais non ou insuffisamment traitée. Dans notre travail, la GNC (29,8%) était la 2^e^ cause d´HTAM, tout comme dans la série de Yao *et al*. [22]. Selon Lip *et al*. [5], les causes secondaires rénales représentaient 39,9% des cas. Sharma *et al*. [23] en Inde ont observé, sur un total de 135 cas d´HTAM, 47 cas (34,8%) de cause secondaire dont 20 cas de cause rénovasculaire (avec 15 cas de maladie de Takayasu) et 19 cas de néphropathie parenchymateuse.

Les inhibiteurs calciques (IC) étaient la classe thérapeutique la plus utilisée en monothérapie (nicardipine) ou en association avec les autres classes d´antihypertenseurs. Selon Herbland *et al*. [12] les IC étaient également en première intention du traitement de l´HTAM respectivement chez 94,6%. Yao *et al*. [23] faisait également le même constat avec les IC utilisés dans 99,2% des cas. Notre attitude était en accord avec celle de la littérature [16, 18] où les inhibiteurs calciques en particulier la nicardipine était la plus indiquée en première intention dans l´HTAM en l´absence de contre-indications. L´association avec les bloqueurs du système rénine angiotensine aldostérone est contre-indiquée à la phase aiguë de l´HTAM. Leur introduction doit se faire à distance de l´épisode aigu. Leurs effets bénéfiques sur la PA mais surtout sur la morbidité et la mortalité cardiovasculaire et rénale ont permis d´améliorer le pronostic de l´HTAM [16].

L´évolution a été favorable dans deux tiers des cas. Lengani *et al*. [13] et Houman *et al*. [14] trouvaient respectivement une mortalité de 27,8% et 36,8% proche des nôtres. A distance de l´hospitalisation, nous avons observé une augmentation progressive du nombre de patients ayant une PA contrôlée et une stabilisation de la fonction rénale. Les facteurs tels que: l´IR terminale; l´hyperkaliémie; l´hyponatrémie, l´anémie ainsi que sa sévérité (p = 0,003) et l´aggravation de l´IR à M1 étaient associés au décès chez nos patients. Dans l´étude de Yao *et al*. [22] portant sur une population de patients avec HTA sévère, l´IR terminale, la rétinopathie hypertensive et l´anémie étaient associées au risque de décès. Shantsila *et al*. [24], dans une étude portant sur le registre de l´HTAM de l´ouest Birmingham ont observé 34% de patients décédés ou en dialyse. Les facteurs prédicteurs étaient l´âge avancé au diagnostic, l´usage antérieur d´antihypertenseurs, la créatinine de base élevée et la protéinurie. Lane *et al*. [25] avaient montré auparavant dans une étude portant sur le même registre que l´âge avancé, la créatinine de base élevée et l´absence de contrôle de la PA étaient associés au risque de décès. Globalement, la survie des patients avec HTAM s´est considérablement améliorée avec l´utilisation de nouveaux médicaments antihypertenseurs [25, 26]. Quant à la survie rénale, la prévalence de l´insuffisance rénale est élevée au cours de l´HTAM [27], comme c´était le cas dans notre étude. Les facteurs prédicteurs d´IRCT après la phase aiguë sont la créatinine élevée et l´absence de contrôle de l´HTA lors du suivi [22, 28]. Selon Gonzalez *et al*. [29], le pronostic rénal de l´HTAM a été amélioré ces dernières années. La protéinurie est un facteur pronostic fondamental pour la survie rénale. Dans notre étude, le pronostic vital de nos patients dépendait plutôt de la sévérité de l´insuffisance rénale ainsi que de ses complications métaboliques.

### Limite de l´étude

Nos résultats ne sauraient être généralisés à l´ensemble des cas d´HTAM hospitalisés dans d´autres services comme la cardiologie et la neurologie. La principale difficulté que nous avons rencontrée était le nombre élevé de dossiers inexploitables. Compte tenu des conditions socioéconomiques précaires de nos patients, ils n´arrivaient toujours pas à honorer les bilans prescrits. En outre, le caractère rétrospectif de notre étude ne nous a pas permis d´avoir toutes les informations pour étudier les différents aspects de l´HTAM en particulier l´évolution à long terme. Les patients avant leur admission dans notre service ont transité le plus souvent soit aux urgences médicales, soit dans une formation sanitaire. Un traitement antihypertenseur était le plus souvent mis en place abaissant les chiffres tensionnels et constituant un biais dans le recrutement de nos patients.

## Conclusion

L´HTA maligne est une situation fréquente. Elle touche l´adulte jeune. Il s´agit dans la majorité des cas de patients hypertendus connus mais irrégulièrement suivis. Ces patients sont admis pour des complications graves dominées dans notre contexte par l´IRC. Et cette IRC au stade avancée expose à de nombreuses complications métaboliques telles que : l´anémie, l´hyperkaliémie et l´hyponatrémie qui étaient les facteurs associés au risque de décès avec une mortalité hospitalière élevée.

### Etat des connaissances sur le sujet

- L´incidence de l´hypertension artérielle maligne est en régression dans les pays développés et elle atteint les sujets jeunes;
- La prévalence de l´hypertension artérielle maligne est élevée dans les pays en voie de développement;
- Les facteurs associés au décès sont: l´âge avancé au diagnostic de l´hypertension, le taux élevé de la créatinine de base et l´usage antérieur d´anti hypertenseurs.

### Contribution de notre étude à la connaissance

- En cas d´insuffisance rénale, l´hyperkaliémie, l´hyponatrémie et l´anémie étaient associées à un mauvais pronostic vital à un mois de l´hypertension artérielle maligne;
- Le pronostic vital immédiat, dans notre contexte, était lié à la sévérité de l´insuffisance rénale et aux troubles métaboliques;
- Le pronostic rénal, dans notre contexte, était sévère avec un taux de 78% d´insuffisance rénale chronique.
